# From Court to Couch: Exercise and Quality of Life after Acute Type A Aortic Dissection

**DOI:** 10.1055/s-0041-1731403

**Published:** 2021-10-05

**Authors:** Selena R. Pasadyn, Eric E. Roselli, Amanda S. Artis, Cassandra L. Pasadyn, Dermot Phelan, Eugene H. Blackstone

**Affiliations:** 1 Aorta Center, Heart and Vascular Institute, Cleveland Clinic, Cleveland, Ohio; 2 Department of Thoracic and Cardiovascular Surgery, Heart and Vascular Institute, Cleveland Clinic, Cleveland, Ohio; 3 Department of Quantitative Health Sciences, Research Institute, Cleveland Clinic, Cleveland, Ohio; 4 Department of Cardiovascular Medicine, Heart and Vascular Institute, Cleveland Clinic, Cleveland, Ohio

**Keywords:** athletics, cardiac rehabilitation, cardiac surgery, physical activity, thoracic aortic disease

## Abstract

**Background** Acute Type A aortic dissection can be physically and mentally stressful with little known about survivors' postrepair activity levels, exercise habits, and quality of life (QOL). This study was aimed to describe pre- and postdissection changes regarding exercise, understand physician recommendations, quantify use of cardiac rehabilitation, and assess QOL in dissection survivors.

**Methods** A total of 295 acute Type A aortic dissection survivors were surveyed about exercise, cardiac rehabilitation, QOL, sexual activity, and posttraumatic stress disorder (PTSD) with 137 (46%) respondents.

**Results** Respondents were less likely to participate in competitive athletics after than before dissection (1/131 [0.76%] vs. 26/131 [20%], *p* [McNemar test] < 0.0001) or lift heavy objects (11/111 [9.9%] vs. 41/111 [37%], *p* < 0.0001). Forty-eight of 132 respondents (36%) did not participate in cardiac rehabilitation. Compared with general population norms, respondents reported lower median QOL physical component scores (40 [26, 51; 15th, 85th percentile], *p* < 0.0001); these were lower in respondents who did not exercise (Hodges–Lehmann [HL; 95% confidence interval (CI)]: –6.8 [–11, –2.4], *p* = 0.002), limited sexual activity (–8.0 [–13, –4.3], *p* = 0.0002), or screened positive for PTSD (–10 [–14, –5.3], *p* = 0.0002). Median mental component scores were similar to general population norms (HL [95% CI]: 55 [34, 61], *p* = 0.24) but were lower among respondents who did not exercise (–4.2 [–7.8, –1.0], *p* = 0.01), limited sexual activity (–5.5 [–10, –1.8], *p* = 0.003), or screened positive for PTSD (–16 [–22, –10], *p* < 0.0001).

**Conclusion** Physicians should prescribe cardiac rehabilitation, encourage appropriate exercise, promote resumption of sexual activity, and identify and treat PTSD after surgery for acute Type A aortic dissection.

## Introduction

Acute Type A aortic dissection is life-threatening with high short- and long-term morbidity and mortality, 1 requiring expeditious surgery. 2 The sudden nature of the dissection causes patients to associate the dissection with coincident activities as precipitating sources of hemodynamic stress. The association of dissection with exercise appears supported by reports of dissection related to blood pressure elevation during resistance training and intense weightlifting. 3 4 Therefore, both dissection survivors and their physicians often express concern regarding exercise despite lack of evidence, supporting the leap from association to causation. This uncertainty and a paucity of data on the exercise habits of dissection patients before and after repair has made it difficult to counsel patients postoperatively. 5

Beyond exercise limitations, these patients have lower self-reported quality of life (QOL) than population norms, particularly their physical component scores. 6 However, not fully explored are factors, such as lack of exercise that may contribute to lower QOL scores. 7 Therefore, in a cohort of patients who survived surgery for acute Type A aortic dissection, we sought to (1) compare level of competitive athletic participation, strength training exercise, and lifting of heavy objects before and after repair; (2) evaluate consistency of physician recommendations regarding exercise after repair; (3) understand use of postoperative cardiac rehabilitation; (4) describe patient-reported physical and mental QOL after repair; and (5) assess the association of QOL with exercise, cardiac rehabilitation, sexual activity, and posttraumatic stress disorder (PTSD).

## Materials and Methods

### Patients

**Fig. 1:**
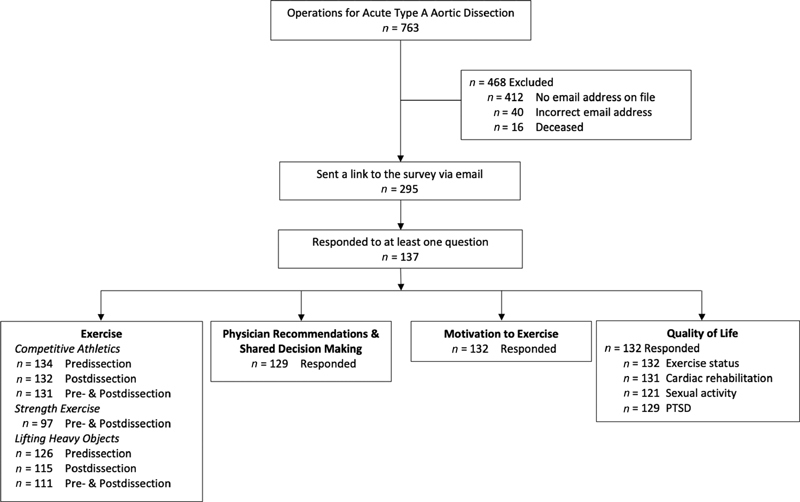
Description of total study population, reasons for exclusion, and number of participants responding to each portion of the survey.

From January 1, 1980 to July 1, 2017, 763 consecutive adults underwent an operation for acute Type A aortic dissection at Cleveland Clinic. An online lifestyle survey about the dissection experience, modified from the survey by Chaddha et al 3 ( Supplementary Table S1 ; available in the online version), 5 was administered to 295 survivors with valid e-mail addresses; 137 (46%) responded ( Fig. 1 ). All survey questions were optional, including patient identifiers. Use of these data for research was approved by the Institutional Review Board (IRB), with patient consent completed at the beginning of the survey.

Among the 137 respondents, the median age at dissection was 57 years and at survey completion was 63 years; the median time between these two time points was 5.8 years ( Table 1 ).

**Table 1:** Characteristics of patients matched with medical records

Variable	*n* a	*n* (%) or median (15th, 85th percentiles)
*Demographics* :		
Sex, female	73	19 (26)
* Race* :		
White	73	65 (89)
Black	73	5 (6.8)
Other	73	1 (1.4)
Age at dissection (y)	115	57 (41, 70)
Age at survey (y)	126	63 (49, 75)
Time from dissection to completion of survey (y)	121	5.8 (1.4, 11)
*Postoperative complications* :		
Permanent stroke	73	2 (2.7)
Reoperation for bleeding/tamponade	73	2 (2.7)
Other noncardiac reoperation	73	3 (4.1)
New-onset postoperative atrial fibrillation	66	25 (38)
New-onset renal failure requiring dialysis	73	3 (4.1)
New-onset renal failure	73	7 (9.6)
Prolonged ventilation	73	24 (33)
* Number of postoperative complications* :	73	
0		31 (42)
1		24 (33)
2		11 (15)
3+		7 (9.6)

a Number available of *n* = 137.

Owing to the ethical concerns raised by the IRB, patients were allowed to remain anonymous. Thus, matching patients to electronic medical records was limited. Of 73 nonanonymous respondents, 54 (74%) were men, 65 (89%) were White, and 5 (6.8%) were Black ( Table 1 ). Postoperative complications were defined according to The Society of Thoracic Surgeons (STS) National Database, Adult Cardiac Surgery (please refer *http://www.sts.org/sts-national-database/database-managers/adult-cardiac-surgery-database/data-collection* ). Of the 73, 31 (42%) had no postoperative complications, 24 (33%) had 1, 11 (15%) had 2, and 7 (9.6%) had ≥3 complications ( Table 1 ).

### Survey Instruments

#### Exercise

The survey included questions about exercise habits before and after dissection repair related to participating in competitive athletics, strength training exercise, and lifting heavy objects; “after dissection” was defined as occurring within 8 weeks prior to responding to the questionnaire ( Supplementary Table S1 ; available in the online version only). Patients were asked if they had been lifting a heavy object when the dissection occurred. Finally, patients were asked if they currently limit the amount of weight they lift, push, or pull, including lifting children. In total, 134 responded to at least one question related to exercise.

#### Physician Exercise Recommendations and Shared Decision-Making

Patients were asked about recommendations by their physicians regarding exercise postdissection. This included questions about restrictions on strength training and aerobic exercise, weightlifting, and maximum effort allowed ( Supplementary Table S1 ; available in the online version only). Patients were asked if they engaged in shared decision-making regarding these exercise recommendations. In total, 129 responded to at least one question related to physician exercise recommendations.

#### Cardiac Rehabilitation

Patients were asked if they participated in cardiac rehabilitation postdissection ( Supplementary Table S1 ; available in the online version only), as well as their motivation to exercise, and if applicable, reasons behind lack of motivation. In total, 132 responded to at least one question about cardiac rehabilitation and motivation to exercise.

#### Posttraumatic Stress Disorder Screening

We utilized a four-question validated survey 8 ( Supplementary Table S2 ; available in the online version only) to screen for PTSD. The survey asks about nightmares, avoidance, being constantly on guard, watchful, easily startled, and feeling numb or detached from others, activities, or surroundings in the past month. It has a sensitivity of 78% and specificity of 87% at a cut-off score of 3 with respect to the gold-standard clinician-administered PTSD scale. 8 Respondents were considered to have a positive PTSD screen if they answered yes to three or all four questions.

#### Quality of Life

The validated Veterans RAND 12-Item Health Survey (VR-12) was used to assess general QOL. 9 Its 12 items correspond to eight physical and mental health domains, including general health perceptions, physical functioning, role limitations owing to physical and emotional problems, bodily pain, energy fatigue, social functioning, and mental health. The items are summarized into a physical and a mental component score based on weights derived from the VR-36, a longer QOL survey administered to 877,775 veterans in the 1999 Large Health Survey of Veteran Enrollees (Veterans Health Study). 10 These two summary component scores explain over 90% of the variance of the VR-36.

VR-12 scores are normalized to the U.S. population using a T-score metric with a mean score of 50 and standard deviation of 10. 11 Norms were established and validated based on the Medical Expenditure Panel Survey, a set of large scale surveys administered to individuals across the United States. 12

Both physical and mental component scores were obtained using a SAS Macro (SAS Institute Inc.; Cary, NC) that imputed scores in the presence of missing response items using the Modified Regression Estimate. This approach uses complete cases to estimate a regression equation where only those present items are used; thus, physical and mental component scores can be calculated regardless of missing items. We obtained 132 physical and mental component QOL scores of which 7 (5.3%) were imputed.

### Interrelationships of Quality of Life and Outcomes

#### Quality of Life and Exercise

There were 132 respondents who had data for both VR-12 and exercise. They were considered “current exercisers” if they answered “yes” to participating in competitive athletics, strength or aerobic exercise, or weightlifting in the past 8 weeks.

#### Quality of Life and Cardiac Rehabilitation

There were 131 respondents who had data for both VR-12 and cardiac rehabilitation. Respondents were considered to have participated in cardiac rehabilitation if they answered “yes” to the question asking about their participation in a program postdissection.

#### Quality of Life and Sexual Activity

There were 121 respondents who had data for both the VR-12 and sexual activity. Respondents were considered to have limited sexual activity if they answered “yes” to the question, “Does aortic dissection limit your current sexual activity?.”

#### Quality of Life and Posttraumatic Stress Disorder

There were 129 respondents who had data for both VR-12 and the PTSD screening survey.

### Data Analysis

Analyses were performed using SAS statistical software (SAS version 9.4; SAS Institute, Cary, NC). Categorical variables are summarized as frequencies and percentages and continuous variables as median (15th and 85th percentiles), congruent with mean ± 1 standard deviation. The Chi-squared test was used to test for association between patients being involved in decisions and postdissection participation in exercise. A two-sided *p* –value of <0.05 was considered statistically significant.

#### Exercise

McNemar tests were used on paired, binary variables; only patients who responded to both before and after dissection questions were included. Exact McNemar tests were used to test each hypothesis because of low cell counts within discordant cells.

#### Quality of Life

One-sample Wilcoxon signed rank tests were used to test if median physical and mental scores were statistically different from the norm of 50. Nonparametric Kruskal–Wallis tests were used to test if median physical and mental scores between the following groups were different: (1) current exercisers versus nonexercisers, (2) those who participated in cardiac rehabilitation versus those who did not, (3) those with postdissection limited sexual activity versus those without, and (4) those with a positive PTSD screen versus negative. To test for differences, we employed Hodges–Lehmann (HL) estimate of location shift and its corresponding 95% confidence interval.

## Results

Because each survey question was optional, number of responses accompany all percentages.

### Exercise

#### Pre- and Postdissection Competitive Athletics

Of 137 respondents, 134 answered questions about predissection competitive athletics. Twenty-eight (21%) participated before their dissection, and 20 (71%) of them participated in two or more sports. The most common sports were baseball (9/28; 32%), football (9/28; 32%), golf (8/28; 29%), basketball (7/28; 25%), and middle-distance running (8/28; 29%); 9 (32%) answered “other.” Of 26 competitive athletes who responded to duration of participation, 10 (38%) participated for >10 years, 8 (31%) for 5 to 10 years, and 8 (31%) for 1 to 5 years.

Of 131 who responded to questions about pre and postdissection competitive athletics, 26 (20%) participated in competitive athletics predissection but only 1 (0.76%) participated postdissection, a cyclist ( *p* < 0.0001 for 131 paired responses).

#### Pre- and Postdissection Strength Exercise

**Fig. 2:**
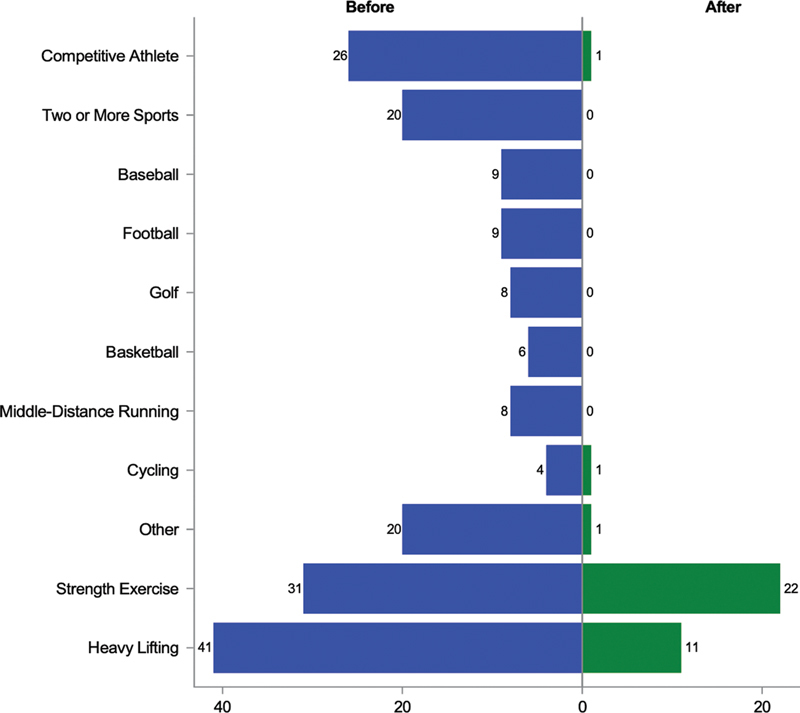
A mirrored histogram of patient participation in competitive athletics, strength exercise, and heavy lifting before and after dissection. The number of participants who participated in the activity before their dissection is represented in blue; the number participating after their dissection is in green.

Ninety-seven (71%) of 137 respondents answered questions about both pre- and postdissection strength exercise. There was no statistical difference between the number of respondents who did strength exercises pre- and postdissection (31 [32%] vs. 22 [23%], *p* = 0.12; Fig. 2 ).

#### Pre-, during, and Postdissection Heavy Object Lifting

When the dissection occurred, 7 (5.3%) of 133 who provided responses said they were lifting a heavy object and 2 (1.5%) were weightlifting. Of those providing a response on how much they were lifting in pounds (lbs), 1 was lifting 10 lbs, 1 was lifting 40 lbs, 1 was lifting 50 lbs, and 2 were lifting 100 lbs.

Of 137 respondents, 111 (81%) answered both pre- and postdissection questions about regularly lifting heavy objects. The proportion who regularly lifted heavy objects decreased from 41 (37%) to 11 (9.9%; *p* < 0.0001; Fig. 2 ).

Of 131 who provided responses, 112 (85%) limited the amount of weight lifted, pushed, or pulled after their aortic dissection. Forty-five (35%) of 127 stated they have not lifted children, and, of these, 16 (36%) reported that this made them feel sad.

### Patient-Reported Physician Exercise Recommendations and Shared Decision-Making

Twenty-one (16%) of 129 providing responses stated that their doctor did not talk to them about exercise and activity after recovering from dissection repair, despite 97 (80%) of 121 responders stating that they wished for specific recommendations about what is safe. Ninety-one (72%) of 126 responders stated their physician was clear in what exercise and daily activities they should and should not do; 71 (78%) stated that their physician placed restrictions on their postdissection exercise, 54 (59%) said their physician restricted strength exercise only, 6 (6.6%) aerobic exercise only, and 11 (12%) both.

Of the 91 providing responses concerning whether their physician was clear in exercise restrictions, 80 (88%) stated the maximum intensity constraint suggested by their physician. Thirty-three (41%) of the 80 said that their maximum was “low,” 45 (56%) said “moderate,” and 2 (2.5%) said “vigorous.” Sixty-five (81%) of these 80 responders completed a question about whether or not their physician gave them a weightlifting limit above which they should not exceed when lifting. Ten (15%) stated there was no limit; the remaining reported a highly variable limit, ranging from 4 to 100 lbs; the most common being 50 lbs, reported by 13 (20%) of 54 respondents.

Of the above 91 responders who stated that their physician was clear in what exercise and daily activities they should and should not do, 84 (92%) reported about their role in shared decision-making. Twenty-seven (32%) stated they were not involved in decisions on their exercise restrictions. Among those with restrictions, 31 (66%) of 47 current exercisers versus 26 (70%) of 37 current nonexercisers were involved in decision-making ( *p* = 0.67).

### Cardiac Rehabilitation and Exercise Motivation

Forty-eight (36%) of 132 who provided responses to the question about cardiac rehabilitation stated that they did not participate in a cardiac rehabilitation program after dissection. Seventy (54%) of 130 responded that they were not motivated to exercise more; of these, 44 (63%) said it was because there were some activities they could no longer do, 35 (50%) said they could not exercise like they used to, 24 (34%) said they were afraid that they would have problems with their aorta in the future, 22 (31%) said they did not know what level of activity was safe, and 25 (36%) responded “other.”

### Quality of Life in Dissection Survivors

#### Overall Quality of Life

**Fig. 3:**
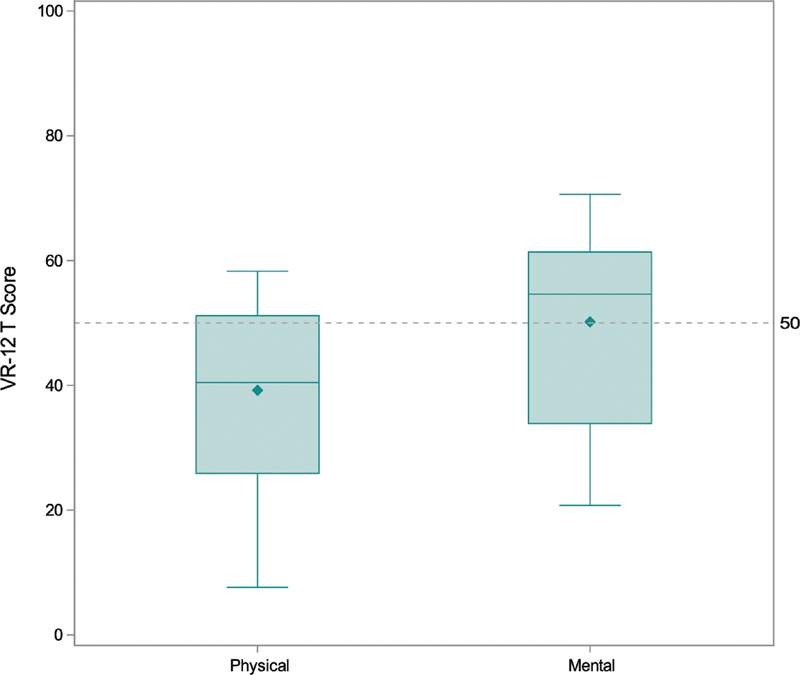
Physical and mental components quality-of-life scores. From bottom to top of the boxplots, the lines represent the 15th percentile, median, and 85th percentile. The whiskers extend to the minimum and maximum component scores. The diamonds represent mean component scores. The dashed line at 50 represents the mean score in the reference population. A total of 132 patients responded for each component. VR-12, the Veterans RAND 12 Item Health Survey.

Of the 132 who responded to QOL questions, median physical component score was 40 (15th, 85th percentiles: 26, 51), significantly lower than 50, the population norm ( *p* < 0.0001). In contrast, the median mental component score was 55 (34, 61), not statistically significantly different from 50 ( *p* = 0.24; Fig. 3 ).

#### Quality of Life and Exercise

**Fig. 4:**
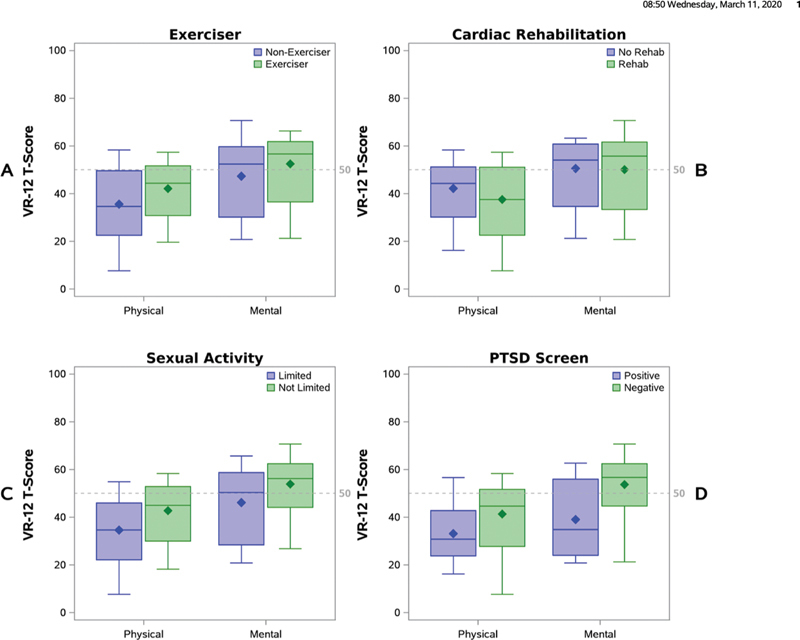
Physical and mental components quality-of-life scores by participation in exercise ( **A** ), cardiac rehabilitation ( **B** ), sexual activity ( **C** ), and scoring positive for PTSD ( **D** ). From bottom to top of the boxplots, the lines represent the 15th percentile, median, and 85th percentile. The whiskers extend to the minimum and maximum component scores. The diamonds represent mean component scores. The dashed line at 50 represents the mean score in the reference population. PTSD, posttraumatic stress disorder; VR-12, Veterans RAND 12 Item Health Survey.

Fifty-eight of 132 who responded to QOL questions (44%) were classified as not current exercisers. Their physical component score was lower than that of respondents who were current exercisers, as was their mental component score ( Table 2 , Fig. 4A ).

**Table 2:** Quality of life and current status after repair of acute Type A aortic dissection

Status	Yes	No	HL (95% CI)	*p-* Value
	Median [15th, 85th percentiles]	Median [15th, 85th percentiles]		
*Not currently exercising* :	*n* = 58	*n* = 74		
Physical QOL score	35 [22, 50]	44 [31, 52]	−6.8 (−11, −2.4)	**0.002**
Mental QOL score	52 [30, 60]	57 [36, 62]	−4.2 (−7.8, −1.0)	**0.01**
*No cardiac rehabilitation* :	*n* = 47	*n* = 84		
Physical QOL score	44 [30, 51]	38 [22, 51]	4.5 (0.11, 8.9)	**0.04**
Mental QOL score	54 [35, 61]	56 [33, 62]	−0.17 (−3.3, 3.6)	0.89
*Current limited sexual activity* :	*n* = 44	*n* = 77		
Physical QOL score	35 [22, 46]	45 [30, 53]	−8.0 (−13, −4.3)	**0.0002**
Mental QOL score	50 [28, 59]	56 [44, 62]	−5.5 (−10, −1.8)	**0.003**
*PTSD* :	*n* = 30	*n* = 99		
Physical QOL score	31 [24, 43]	45 [28, 52]	−10 (−14, −5.3)	**0.0002**
Mental QOL score	35 [24, 56]	57 [45, 62]	−16 (–22, −10)	**<0.0001**

Abbreviations: CI, confidence interval; HL, Hodges–Lehmann; P, percentile; PTSD, posttraumatic stress disorder; QOL, quality of life.

#### Quality of Life and Cardiac Rehabilitation

One hundred and thirty-one of 132 who responded to QOL questions (99%) replied to our question about participation in rehabilitation; 84 (64%) affirmed participating. Respondents who did not participate had higher physical component scores than those who did but their mental component scores were similar ( Table 2 , Fig. 4B ).

#### Quality of Life and Sexual Activity

Of 132 who responded to QOL questions, 121 (92%) answered a question about their current sexual activity; 44 (36%) said it was limited. Both physical and mental component scores were lower in respondents who reported current limited sexual activity than in those who did not limit their sexual activity ( Table 2 , Fig. 4C ).

#### Quality of Life and PTSD

Of 129 who responded to both QOL and PTSD questions, 30 (23%) screened positive for PTSD. These patients had lower physical and mental component scores compared with those who screened negative ( Table 2 , Fig. 4D ).

## Discussion

### Principal Findings

Predissection, respondents participated in competitive athletics, strength exercise, and lifting heavy objects; these activities were substantially reduced after repair of their acute dissection. Despite a majority desiring guidance, there was large variation in physician recommendations for exercise and lifting postdissection. Over one-third of respondents did not participate in cardiac rehabilitation after dissection repair. Respondents had lower physical, but not mental, QOL scores than the general population. Patients who were nonexercisers reported limited sexual activity. Those with a positive PTSD screen had lower physical and mental QOL scores.

### Decline in Exercise Postdissection

Exercise is particularly relevant to patients after surviving aortic dissection, as it promotes cardiovascular health through its positive effects on heart rate and blood pressure. It also has a role in promoting a healthy mental state and overall improved QOL. 13 Engaging in regular exercise lowers resting systolic blood pressure by 3 to 8 mm Hg over time, and this is associated with a lower risk of future aortic complications. 14 However, our findings align with those of Chaddha et al 15 who found that the proportion of Type A dissection survivors, who engaged in no structured physical activity, increased from 17 to 24% after the event.

### Variable Patient-Reported Physician Recommendations and Shared Decision-Making

As we found, physicians have been reported to be unclear about exercise and sport recommendations after Type A aortic dissection repair. Chaddha et al 15 showed that although 58% of providers told their patients to monitor blood pressure and heart rate during physical activity, there was inconsistency about what constituted a safe upper limit. As we found in our study (80%), Chaddha et al 15 found that the majority of their patients (71%) wished for specific recommendations about safe activities postdissection.

Currently, it is thought that age-appropriate light-to-moderate dynamic exercise should be safe and feasible. 16 17 18 Exertion levels of “fairly light” to “somewhat hard” in activities such as brisk walking or cycling are accepted. With weight training, light-to-moderate lifting, limiting the amount of weight used per set, and stopping before volitional fatigue and Valsalva maneuver is recommended. 18 These recommendations are based on expert consensus; however, there is little evidence to support more quantitative and standardized recommendations.

It has been shown that shared decision-making with patients improves adherence to physician recommendations. 19 20 21 Almost one-third of current study respondents were not involved in the decision process of creating an exercise plan. However, we did not find that patient investment in their plan of care changed levels of exercise. This finding suggests that patients may be looking to their physician for clearer exercise guidance.

### Utility of Cardiac Rehabilitation

The benefit of cardiac rehabilitation has been demonstrated in patients who survive myocardial infarction and cardiac surgery, but evidence supporting cardiac rehabilitation after aortic dissection is limited. In a study of 33 survivors of acute Type A aortic dissection, Corone et al 22 found that mean maximum physical work capacity of those who used cardiac rehabilitation increased from 63 to 92 Watts/min at the end of the intervention. Despite this evidence and the general appreciation that these patients with chronic aortic disease may benefit from cardiac rehabilitation, our findings demonstrated that one-third of patients did not engage. Interestingly, we found that those who participated in cardiac rehabilitation had lower physical component QOL scores. It is possible that the patients who recommended for and pursued cardiac rehabilitation were more functionally impaired at baseline.

### Quality of Life in Aortic Dissection Survivors

Self-reported physical, mental, and social wellbeing has become an important component in assessing personal health. 23 24 Lower QOL has been previously documented in aortic dissection survivors. 25 Adam and colleagues 6 studied 188 survivors following repair of acute Type A aortic dissection finding lower physical and mental component scores compared with norms. In their study and that of Jussli-Melchers and colleagues, 25 there was a more pronounced departure from norms in the physical component score than in the mental component score, as we found.

#### Quality of Life and Exercise

Physical activity is associated with higher QOL scores across many populations. The relationship between the two is less well studied in dissection patients. Adam and colleagues 6 found that patients who engaged in sports before their dissection had higher QOL scores after dissection repair, and we replicated this finding.

#### Quality of Life and Sexual Activity

Older adults who engage in sexual activity tend to have higher QOL scores and greater psychological wellbeing. 26 Despite the benefits, many individuals are fearful to resume sexual activity after dissection; this was associated with lower QOL scores in our respondents. There is no evidence to suggest that engaging in sexual activity increases risk for subsequent aortic events or progression of disease.

#### Quality of Life and Posttraumatic Stress Disorder

PTSD is a chronic and disabling psychological disorder that can develop after exposure to highly stressful events characterized by actual or threatened harm to the self or others. PTSD is associated with lower QOL, and this is also true postdissection as we found. The fearful, avoidant and numbing symptoms from the life-threatening dissection event can impair patient psychosocial functioning, likely leading to lower QOL.

### Limitations

This study emanates from a single institution with a large volume of aortic dissections which may limit generalizability. Our survey was voluntary and administered via e-mail; thus, the sample is self-selected and limited in power. Also, the cross-sectional nature of this follow-up limited our ability to track longitudinal changes and limits conclusions to associations rather than inferences of causality. However, well over 40% of respondents completed the survey, and important insights can guide patient care and further investigation.

### Clinical Implications

Given the benefits of physical activity, but its marked reduction in dissection survivors, physicians should address exercise at follow-up visits. Physicians should inquire about predissection activity level, so they can encourage resumption of some of the same practices, or modified versions of them after the event. For example, it is likely safe for a golfer to return to sport. Until data-driven exercise guidelines are established, physicians will need to devise individualized plans with patients based on their capacity, considering several factors such as predissection abilities, blood pressure, mobility, and age. Physicians should encourage patients to participate in cardiac rehabilitation, so their patients can increase their exercise abilities and work capacity.

To bring QOL scores as close as possible to those of population norms, it is important to identify actionable changes that can be made in the lives of dissection survivors. Not exercising, limiting sexual activity, and having a PTSD diagnosis are associated with lower physical and mental QOL. Therefore, physicians should encourage exercise. Sexual activity for both men and women allows for emotional and physical intimacy, an important component of health, and a frank discussion with dissection survivors, reassuring them that resuming sexual activity is safe, should be encouraged. Physicians should also screen for PTSD, and patients screening positive should be referred for definitive diagnosis and treatment when it is identified. Trauma-focused psychotherapy or pharmacotherapy may be indicated.

## Conclusion

Aortic dissection survivors face physical and mental obstacles associated with the life-long burden of having a chronic condition after successful surgery performed in the acute phase. Physicians are in a position to promote comprehensive, life-long care of these patients. By encouraging exercise, discussing sexual activity practices, and addressing and treating PTSD and mental and physical QOL may be improved for acute Type A aortic dissection survivors.
