# Supplementary material for: From Court to Couch: Exercise and Quality of Life after Acute Type A Aortic Dissection
Source: Aorta (Stamford). 2021 Oct 5;9(5):171–9. doi: 10.1055/s-0041-1731403 (PMC8654512; doi:10.1055/s-0041-1731403)
Supplement: Supplementary file 1 — Supplementary Material [file 10-1055-s-0041-1731403-s200047.pdf]

Supplementary Table S1 Survey: Available Questions and Response Options[22–24]

Question	Response Option
Answer the following with regard to your dissection:	
What were you doing when it started?<br>Please choose one option.	Coughing<br>Exercising<br>Having sex<br>Lifting a heavy object<br>Sleeping<br>Stress<br>I forget<br>Other
If lifting a heavy object, how many pounds were you lifting?	
Did you do competitive athletics before the dissection?	Yes or No
If Yes, what activities did you do?	Baseball<br>Basketball<br>Body building<br>Competitive weight lifting<br>Cross fit<br>Cycling<br>Field events<br>Football<br>Golf<br>Gymnastics<br>Hockey<br>Lacrosse<br>Long-distance running<br>Middle-distance running<br>Rugby<br>Soccer<br>Sprint-distance running (<400 m)<br>Swimming<br>Tennis<br>Triathlon (iron man)<br>Triathlon (Olympic/sprint)<br>Ultradistance running<br>Volleyball<br>Other
Outside of competitive athletics, pick what type of activity you did consistently (select all that apply):	Aerobic exercise (e.g., running, cycling)<br>Strength exercise (e.g., weightlifting, resistance training)
Did you lift heavy objects on a regular basis?	Yes or No
Answer the following with regard to the last 8 weeks:	
Are you doing competitive athletics now?	Yes or No
If Yes, what activities did you do?	Baseball<br>Basketball<br>Body building<br>Competitive weight lifting<br>Cross fit<br>Cycling<br>Field events<br>Football<br>Golf<br>Gymnastics<br>Hockey<br>Lacrosse<br>Long-distance running<br>Middle-distance running

 © 2021. The Author(s).

Supplementary Table S1 (*Continued*)

Question	Response Option
	Rugby<br>Soccer<br>Sprint-distance running (<400 m)<br>Swimming<br>Tennis<br>Triathlon (iron man)<br>Triathlon (Olympic/sprint)<br>Ultradistance running<br>Volleyball<br>Other
Outside of competitive athletics, pick what type of activity you do consistently (select all that apply):	Aerobic exercise (e.g., running, cycling)<br>Strength exercise (e.g., weightlifting, resistance training)
Do you lift heavy objects on a regular basis?	Yes or No
Does your aortic dissection motivate you to exercise more?	Yes or No
If no, why?	There are some activities I can no longer do<br>I can't exercise like I used to<br>I am afraid I will have problems with my aorta in the future<br>I don't know what level of activity is safe for me<br>I am afraid to exercise because I might cause further damage to my aorta<br>I am afraid to exercise because I might die from my aortic disease<br>Other
Did your doctor talk to you about exercise and activity?	Yes or No
Was your doctor clear in what exercise and daily activities you should and should not do?	Yes or No
On which of the following did your doctor place a restriction (check all that apply):	Aerobic exercise (e.g., running, cycling)<br>Strength exercise (e.g., weightlifting, resistance training)
Indicate the maximum intensity your doctor allowed:	Low<br>Moderate<br>Vigorous
Was there a weight limit that you were advised not to go above when lifting?	Yes or No
What was that weight (lbs)?	
Were you involved in the decision-making on those restrictions?	Yes or No
Do you wish there were specific recommendations about what is safe for aortic dissection patients?	Yes or No
Did you go through rehabilitation after your aortic dissection?	Yes or No
Since your aortic dissection, do you limit the amount of weight you lift, push, or pull?	Yes or No
Can you lift up children?	Yes or No
If no, how does that make you feel?	It does not affect my life<br>It makes me feel sad<br>Other
Does your diagnosis of aortic dissection limit your current sexual activity?	Yes or No

 © 2021. The Author(s).

Supplementary Table S2 Posttraumatic Stress Disorder (PTSD) Screening Questions[a]

Question	Patient Response
With regard to your dissection (please circle the following):<br>Have you had nightmares about it or thought about it when you did not want to?	Yes or No
Have you tried hard not to think about it or went out of your way to avoid situations that reminded you of it?	Yes or No
Were you constantly on guard, watchful, or easily startled after your aortic dissection?	Yes or No
Did you feel numb or detached from others, activities, or your surroundings after your aortic dissection?	Yes or No

[a]Demonstration of how the 4 Item Primary Care-PTSD screening tool was worded on the survey administered to all acute type A dissection patients.[19,25] Patients were asked the questions specifically "with regard to your dissection" so the PTSD identified was attributed only to the dissection event.

 © 2021. The Author(s).
